# Pallidal Deep Brain Stimulation in Dystonia: Investigating Differential Response by Dystonia Distribution

**DOI:** 10.5334/tohm.1175

**Published:** 2026-06-26

**Authors:** Sushuma Yarlagadda, Margi Patel, Sheila Rajagopalan, H. A. Jinnah, Stewart A. Factor, Svjetlana Miocinovic, Laura M. Scorr

**Affiliations:** 1 Emory University Department of Neurology, Atlanta, GA, USA; 2 Emory University School of Medicine, Atlanta, GA, USA; 3 Baylor University Medical Center, Dallas, TX, USA; 4 Emory University Department of Human Genetics, Atlanta, GA, USA; 5 Emory University Department of Pediatrics, Atlanta, GA, USA; 6 Jean and Paul Amos Parkinson’s Disease and Movement Disorder Program, Atlanta, GA, USA

**Keywords:** Dystonia, deep brain stimulation, globus pallidus

## Abstract

**Background:**

Dystonia is characterized by involuntary intermittent or sustained abnormal movements or postures. Deep brain stimulation (DBS) is an effective treatment, and response has been shown to vary across body regions but literature depicting this pattern is conflicting. Our study analyzes dystonia response to DBS by body distribution using the Burke-Fahn-Marsden Rating Scale motor (BFMRS-M) score and the Global Dystonia Rating Scale (GDRS).

**Methods:**

We reviewed standardized videos of patients with isolated non-acquired dystonia obtained at baseline and post-DBS of unilateral or bilateral globus pallidus interna between 2008 and 2020. BFMRS-M and GDRS were scored using blinded assessment by two movement disorders neurologists. Mean baseline and post-DBS scores were compared using a paired t-test.

**Results:**

Ten male and 10 female patients were included, with average age at surgery 49 years. Post-DBS total BFMRS-M scores demonstrated improvement (20.5 ± 3.6 vs 11.9 ± 2.6) and there was significant improvement in neck sub-scores (4.9 ± 0.6 vs 2.5 ± 0.4). All patients demonstrated an improved total GDRS score post-DBS (21.6 ± 3.7 vs 11.1 ± 2.1), with significant improvements in GDRS sub-scores of the neck (5.9 ± 0.6 vs 2.6 ± 0.4) and shoulder & proximal arms (4.7 ± 1.1 vs 1.6 ± 0.5).

**Discussion:**

Efficacy of DBS varies by distribution of dystonia and should be considered in treatment planning discussions. GDRS is a more sensitive outcome measurement for dystonia compared to BFMRS-M and should be more routinely integrated into studying treatment response.

**Highlights:**

This study shows that pallidal deep brain stimulation yields differential improvement across dystonia body regions, with greatest benefit in the neck and proximal arms. These results highlight that the Global Dystonia Rating Scale is more sensitive to regional outcomes compared to the commonly utilized Burke-Fahn-Marsden Rating Scale.

## Introduction

Dystonia is a neurological disorder characterized by involuntary sustained or intermittent abnormal, often repetitive, movements or postures [1]. This disorder can manifest across any body region, including face, neck, trunk, and extremities, leading to substantial functional impairment and diminished quality of life for affected individuals [2,3]. Dystonia can be classified according to etiology and clinical characteristics. Etiological classification includes genetic, acquired, or idiopathic dystonia. Clinical characteristics used to classify dystonia and guide treatment are age at onset, temporal pattern, whether additional movement disorders are present (combined dystonia) or absent (isolated dystonia), and body distribution. Body distribution can be further classified into focal, multifocal, segmental, hemidystonia, and generalized [1].

Deep brain stimulation (DBS) is an important treatment approach for patients with dystonia who failed pharmacotherapy, failed botulinum toxin therapy, or were not a candidate for botulinum toxin due to widespread dystonia [4,5,6]. While the exact mechanisms underlying DBS are not yet fully elucidated, emerging evidence suggests it acts to improve aberrant neural activity [6,7,8,9]. The target of stimulation in dystonia is typically the globus pallidus internus (GPi) or the subthalamic nucleus, but in some cases the thalamus may be more appropriate [10]. Studies have reported generally positive outcomes in the overall efficacy of DBS for dystonia, with improvements in motor function, alleviation of pain, and overall quality of life [6,7,8,9].

Several knowledge gaps and areas of debate persist regarding DBS response in dystonia. Notably, there have been few studies comparing the effectiveness in various body regions, making it difficult to provide patients with an expected pattern of response. Some studies have demonstrated that axial dystonia (e.g. blepharospasm, cervical dystonia, and laryngeal dystonia) shows less improvement with DBS than limb dystonia [11,12,13,14,15,16,17,18]. However, another study of 11 patients with idiopathic segmental dystonia revealed greater improvement in axial symptoms than in distal upper or lower limbs [19]. Among patients with isolated inherited and idiopathic dystonia, the generalized, segmental, and cervical subtypes have demonstrated the greatest improvement with DBS, while focal and multifocal dystonia show less improvement in total severity scores [20,21,22,23]. Thus, the existing literature is limited by conflicting data, small sample sizes, and variable inclusion criteria.

DBS literature typically utilizes the Burke-Fahn-Marsden Rating Scale motor (BFMRS-M) scores to evaluate dystonia [24,25]. The BFMRS-M attributes points by body region for clinical severity and provoking factors on a scale of 0 to 4, then assigns a weighted total score (0.5 for eyes, mouth, neck; 1.0 for all other regions) [26]. There are widely recognized limitations of the BFMRS-M such as the weighted calculations leading to distortion, decreased sensitivity, and decreased discriminative capability [27]. A more recently developed metric, the Global Dystonia Rating Scale (GDRS), rates 10 body regions on a scale of 0 to 10 based on clinical severity [28]. Recent studies suggest that GDRS may be better suited than BFMRS-M for evaluating individual body regions, because it provides more granular regional detail and avoids weighted scoring [27]. Despite this, BFMRS-M remains the most commonly used outcome measurement in dystonia studies [29].

The primary objective of this report is to quantify the response of multiple body regions to GPi DBS in isolated non-acquired dystonia. By better defining this differential response, we seek to provide valuable insights to better inform prognostic discussions and treatment decisions with patients being evaluated for DBS therapy. The secondary objective is to evaluate this response using both BFMRS-M and GDRS metrics to examine the comparative utility and limitations of each scale. By integrating these objectives, this work aims to strengthen the existing methodological framework for assessing therapeutic response to DBS in dystonia.

## Methods

### Subjects

**Figure 1:**
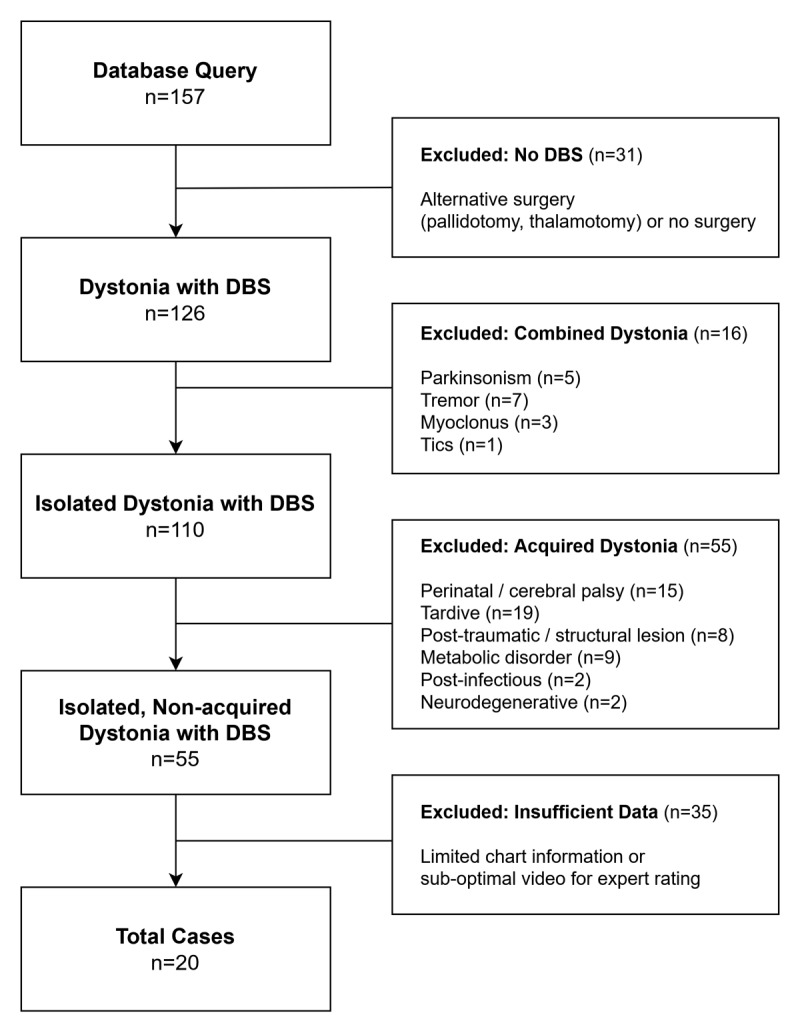
**Patient selection**. Flow diagram illustrating inclusion and exclusion criteria for cohort selection.

In the DBS program at Emory University, patients who provide informed consent are included in a database whose use for research is approved by the local IRB and includes demographic data, clinical characteristics, and treatment response. Clinical data also includes a collection of pre-operative and post-operative standardized video assessments with rating scales. A flow diagram of patient selection is shown in Figure 1. The database was queried, and patients were identified with isolated genetic or idiopathic dystonia who received DBS implantation targeting the GPi between 2008 and 2020 [1]. Patients were excluded if they did not undergo DBS, if they had combined dystonia or acquired dystonia, if their medical chart was inaccessible or with limited information, or if their videos did not have sufficient duration or quality to allow clinical rating of all body parts [1].

### Video Review Protocol

Using the standardized videos from the database, two fellowship-trained movement disorders neurologists (MP and SAF) separately completed a randomized evaluation by scoring both BFMRS-M and GDRS at baseline and post-DBS for each patient, and scores were averaged. Expert raters were blinded to patient identity and patient order as well as timepoint of pre- or post-DBS. If sub-scores differed by ≥2 or a total score differed by ≥3, a third movement disorder neurologist’s score was included in the average (SM).

### Data Analysis

For scores of total dystonia and each body region, mean baseline BFMRS-M scores were calculated and compared to the mean post-DBS counterparts using a two-tailed paired t-test. Similarly, mean baseline GDRS total scores and body region sub-scores were compared to the mean post-DBS counterparts using a two-tailed paired t-test. Statistical significance was determined with both an uncorrected p-value < 0.05 and an adjusted p-value that applied Bonferroni correction to account for multiple comparisons (p < 0.00625 for BFMRS-M scores which had 8 t-tests and p < 0.0045 for GDRS scores which had 11 t-tests). While Bonferroni correction was applied to control for type I error, uncorrected p-values were also included to preserve interpretability, as strict correction may increase the risk of type II error. For each comparison between mean baseline and mean post-DBS score, standard error was calculated.

Patient-level analysis was completed using BFMRS-M and GDRS. Patients were stratified into the following groups based on the degree of change between baseline and post-DBS scores: >50% improvement, <50% improvement, worse, or no change. The number of patients in each of these categories for each body region were tallied. Patients with no dystonia in a particular body region (baseline sub-score 0 and post-DBS sub-score 0) were excluded from analysis when pertaining to that body region.

## Results

Patient characteristics are shown in Table 1. Of the total 20 patients, 10 were male and 10 were female. Among them, 8 (40%) had generalized dystonia, 9 (45%) segmental dystonia, 2 (10%) hemidystonia, and 1 (5%) focal cervical dystonia. Six patients had genetic results available with 3 TOR1A, 1 THAP1, 1 negative DYT1 gene test, and 1 negative dystonia panel. The average age of dystonia onset was 35 ± 20 (range 5–70) years and the average age at the time of surgery was 49 ± 19 (range 10–72) years. The average postoperative follow-up interval was 14 ± 7 (range 6–30) months. DBS characteristics and stimulation parameters are shown in Table 2. The two patients with hemidystonia both had left GPi implantation while all other patients had bilateral GPi implantation.

**Table 1:** Patient characteristics.


PATIENT	GENDER	AGE AT DYSTONIA ONSET (YEARS)	DYSTONIA PHENOTYPE	GENETIC TESTING	AGE AT TIME OF SURGERY (YEARS)	MEDICATIONS (AT TIME OF SURGERY)	SURGERY TO FOLLOW-UP (MONTHS)

1	M	6	Generalized	TOR1A, GAG946 del	11	Artane 2 mg TID	11.5

2	F	6	Generalized	TOR1A, GAG946 del	10	Baclofen 15 mg TID Trihexyphenidyl 30 mg TID Levodopa 300 mg/day	28.2

3	M	9	Generalized	TOR1A, c.907_909delGAG	41	Diazepam 10 mg TID	6.6

4	M	11	Generalized	THAP1, c.256A>G	33	Clonazepam 0.5 mg weekly	10.6

5	F	41	Generalized	Not available	48	Botulinum injections Clonazepam 1 mg nightly Cyclobenzaprine 10 mg TID	21.4

6	M	53	Segmental	Not available	58	Botulinum injections Lorazepam 2 mg QID	8.0

7	F	39	Generalized	Not available	59	Botulinum injections Trihexyphenidyl 2 mg TID Primidone 100 mg nightly	9.2

8	M	5	Hemidystonia	Negative DYT1 GAG946 del	18	None	19.9

9	M	53	Segmental	Not available	57	Botulinum injections Trihexyphenidyl 2 mg TID	14.0

10	M	31	Segmental	Not available	65	None	7.6

11	M	43	Hemidystonia	Negative Dystonia Panel	61	Baclofen 20 mg TID	12.3

12	F	55	Generalized	Not available	66	Flexeril 10 mg TID Propranolol 40 mg BID	12.9

13	F	33	Generalized	Not available	59	Botulinum injections Propranolol 40 mg/day Baclofen 40 mg BID Primidone 50 mg BID Clonazepam 1 mg nightly	12.7

14	F	58	Segmental	Not available	64	Botulinum injections Clonazepam 1 mg TID Trihexyphenidyl 2 mg TID	30.0

15	F	46	Segmental	Not available	66	Botulinum injections Trihexyphenidyl 2 mg TID Clonazepam 1 mg TID	5.8

16	M	20	Segmental	Not available	47	Botulinum injections Clonazepam 1 mg BID	16.0

17	F	70	Segmental	Not available	72	Diazepam 5 mg PRN Clonazepam 1 mg nightly Cyclobenzaprine 10 mg PRN	16.0

18	M	35	Segmental	Not available	37	Botulinum injections	13.6

19	F	56	Segmental	Not available	59	Trihexyphenidyl 1 mg TID Baclofen 10 mg TID	14.9

20	F	25	Focal	Not available	57	None	16.6


**Table 2:** Patient-level DBS characteristics and stimulation settings. Lead coordinates describe the bottom contact unless otherwise indicated. Stimulation settings are at the time of the patient’s video used for post-DBS scoring.


PATIENT	MANUFACTURER	TARGET LATERALITY	DIRECTIONAL LEADS	LEFT LEAD COORDINATES (X, Y, Z)	RIGHT LEAD COORDINATES (X, Y, Z)	LEFT STIMULATION SETTINGS	RIGHT STIMULATION SETTINGS

1	Boston Scientific	Bilateral	Yes	–17.39, 1.75, –4.82	20.59, 2.27, –4.32	C+ 3–(30%) 4–(70%) 3.5 mA, 90 µs, 130 Hz	13+ 11–(50%) 12–(50%) 1.5 mA, 90 µs, 130 Hz

2	Medtronic	Bilateral	No	–20.90, –0.80, –6.70	21.5, –0.8, –4	0+ 2–, 3.5 V, 60 µs, 80 Hz 1200 Ω, 3.0 mA	C+ 10–, 4.5 V, 60 µs, 80 Hz 930 Ω, 4.1 mA

3	Boston Scientific	Bilateral	Yes	–19.85, 5.64, –2.17	21.89, 4.92, –4.09	C+ 1–, 4.7 mA, 60 µs, 130 Hz	C+ 2– 4.7 mA, 60 µs, 130 Hz

4	Medtronic	Bilateral	No	–20.43, 1.43, –3.48	23.77, 2.46, –5.02	C+ 2–, 2 V, 120 µs, 130 Hz 994 Ω, 2.0 mA	8+ 10–, 2.6 V, 150 µs, 130 Hz 1793 Ω, 1.4 mA

5	Medtronic	Bilateral	No	–19.85, 2.13, –3.84	21.47, –0.06, –4.08	C+ 1– 2–, 3.6 V, 90 µs, 130 Hz 814 Ω, 4.4 mA	0+ 3+ 1–, 4.6 V, 90 µs, 150 Hz 776 Ω, 5.9 mA

6	Medtronic	Bilateral	No	–23.71, 0.53, –3.69	22.80, 2.28, –2.53	C+ 1–, 3.5 V, 120 µs, 135 Hz 1121 Ω, 3.3 mA	C+ 9–, 3.5 V, 120 µs, 135 Hz 981 Ω, 3.8 mA

7	Medtronic	Bilateral	No	–20.00, –0.10, –2.80	19.40, –0.80, –2.20	C+ 2–, 3.6 V, 60 µs, 140 Hz 1173 Ω, 3.1 mA	11+ 9– 10–, 3.4 V, 90 µs,140 Hz 1739 Ω, 2.0 mA

8	Medtronic	Left	No	Lead 1: –20.14, –0.45, –0.34 Lead 2: –20.61, 1.30, –2.22	N/A	Lead 1: C+ 9–, 2.7 V, 90 µs, 90 Hz 1164 Ω, 2.3 mA Lead 2: 8+ 11+ 9–, 3.4 V, 90 µs, 90 Hz 1133 Ω, 3.0 mA	N/A

9	Medtronic	Bilateral	No	–22.33, 1.28, –3.37	23.35, 0.47, –3.13	C+ 1–, 5.6 V, 60 µs, 70 Hz 1121 Ω, 5.1 mA	C+ 2–, 4.8 V, 90 µs, 125 Hz 1505 Ω, 3.4 mA

10	Boston Scientific	Bilateral	Yes	–22.77, 3.78, –1.98	23.35, 4.75, –1.95	C+ 2–(50%) 3(50%) 4.6 mA, 70 µs, 130 Hz	C+ 9–, 4.5 mA, 100 µs, 130 Hz

11	Medtronic	Left	No	–22.8, 1.1, –3.6	N/A	1+ 3– 2–, 4 V, 90 µs, 160 Hz	N/A

12	Medtronic	Bilateral	No	–19.30, 0.00, –1.70	19.90, 0.10, –2.40	1+ 2–, 4.4 V, 120 µs, 90 Hz 1385 Ω, 3.3 mA	C+ 1–, 2.8 V, 120 µs, 90 Hz 1111 Ω, 2.6 mA

13	Boston Scientific	Bilateral	Yes	–19.98, 1.47, –3.76	20.84, 2.30, –1.89	1+ 2–(65%) 3–(35%) 4.9 mA, 100 µs, 174 Hz	C+ 11–, 3.6 mA, 60 µs, 130 Hz

14	Medtronic	Bilateral	No	–20.74, –0.39, –5.04	22.06, 0.42, 0.25	C+ 1– 2–, 2.54 V, 90 µs, 130 Hz 1105 Ω, 2.6 mA	0+ 1–, 3 V, 90 µs, 130 Hz 1227 Ω, 2.1 mA

15	Medtronic	Bilateral	No	–20.54, –0.08, –0.74	20.50, –1.35, –3.22	C+ 2–, 3.5 V, 90 µs, 80 Hz 1217 Ω, 2.9 mA	C+ 1–, 3.8 V, 60 µs, 80 Hz 1517 Ω, 2.6 mA

16	Medtronic	Bilateral	No	–20.80, –0.40, –4.50	20.60, 0.20, –3.40	1+ 2–, 3.5 V, 90 µs, 70 Hz	3+ 2–, 3.9 V, 90 µs, 90 Hz

17	Medtronic	Bilateral	No	–20.00, –1.00, –2.00	21.00, –0.50, –2.50	0+ 1–, 4.2 V, 90 µs, 135 Hz 1026 Ω, 4.0 mA	3+ 1–, 3.9 V, 90 µs, 135 Hz 2063 Ω, 1.9 mA

18	Medtronic	Bilateral	No	–20.95, 1.24, –2.67	20.41, 2.45, –5.80	C+ 2–, 3.3 V, 60 µs, 130 Hz 1486 Ω, 2.3 mA	C+ 10–, 3.5 V, 60 µs, 130 Hz 1199 Ω, 2.9 mA

19	Medtronic	Bilateral	No	–18.20, 3.87, –0.78*	19.47, 2.40, 0.04°	C+ 2–, 4 V, 210 µs, 60 Hz 1231 Ω, 3.3 mA	C+ 10–, 4 V, 210 µs, 60 Hz 1142 Ω, 3.5 mA

20	Medtronic	Bilateral	No	–18.72, 4.99, –1.77	19.75, 4.78, –4.30	C+ 2–, 3.2 V, 120 µs, 130 Hz 1152 Ω, 2.8 mA	C+ 1–, 3.4 V, 120 µs, 130 Hz 839 Ω, 4.0 mA


*C2 contact coordinates (bottom contact coordinates not available).

**Figure 2:**
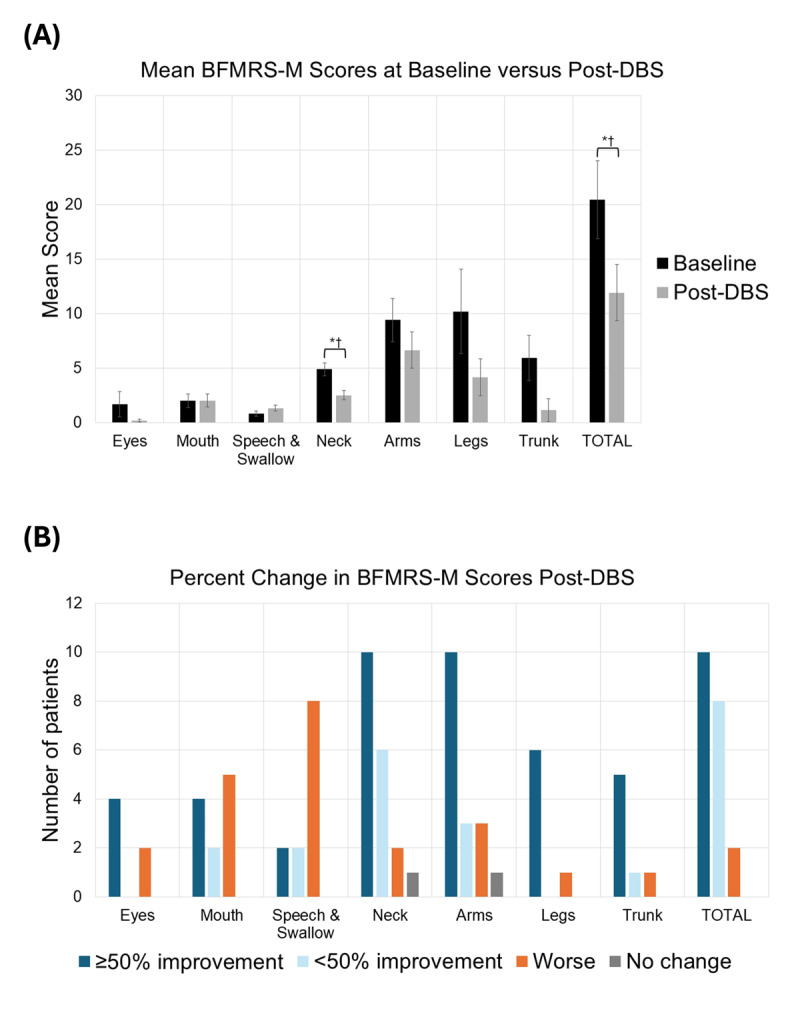
**Response to DBS by dystonia body region using BFMRS-M. (A)** Mean BFMRS-M scores by body region at baseline versus post-DBS. Error bars depict standard error. * Depicts statistical significance with Bonferroni-corrected p < 0.00625. **(B)** Percent change in BFMRS-M scores by body region post-DBS as defined by >50% improvement, <50% improvement, worse score, or no change.

Patient-level BFMRS-M scores for each body region at baseline and post-DBS are shown in Supplemental Table 1. We compared mean baseline BFMRS-M scores to mean post-DBS BFMRS-M scores by body region, as shown in Figure 2A. Using the Bonferroni-corrected p-value < 0.00625, there was a statistically significant improvement in patients with neck dystonia by 49.0% (4.9 ± 0.6 vs 2.5 ± 0.4, p = 0.0007) and total dystonia by 42.0% (20.5 ± 3.6 vs 11.9 ± 2.6, p = 0.0030). Figure 2B shows patient-level data on degree of change from baseline BFMRS-M to post-DBS. In the following body regions, there were more patients who demonstrated improvement compared to patients with no change or worse post-DBS sub-scores: eyes (4 out of 6), neck (16 out of 19), arms (13 out of 17), legs (6 out of 7), and trunk (6 out of 7). Out of the 12 patients with speech & swallow dysfunction, 8 had worse post-DBS sub-scores. Out of the 11 patients with mouth dystonia, 5 had worse post-DBS sub-scores.

**Figure 3:**
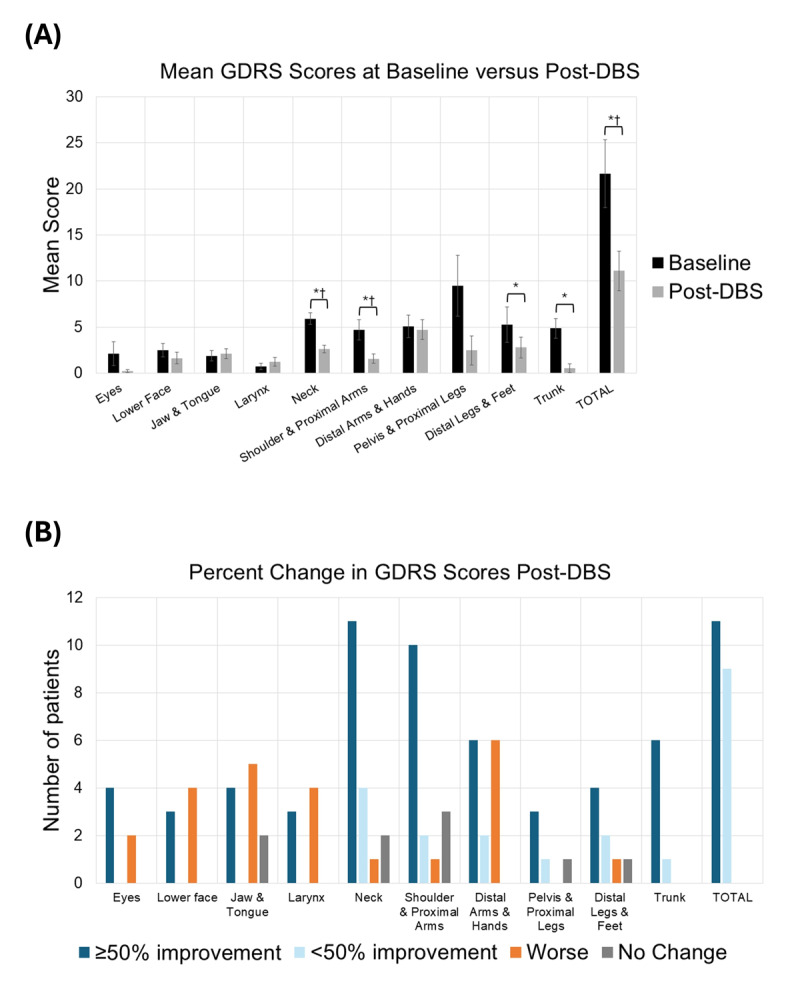
**Response to DBS by dystonia body region using GDRS. (A)** Mean GDRS scores by body region at baseline versus post-DBS. Error bars depict standard error. * Depicts statistical significance with Bonferroni-corrected p < 0.0045. **(B)** Percent change in GDRS scores by body region post-DBS as defined by >50% improvement, <50% improvement, worse score, or no change.

Patient-level GDRS scores for each body region at baseline and post-DBS are shown in Supplemental Table 2. Figure 3A shows mean baseline GDRS scores compared to mean post-DBS GDRS scores. Using the Bonferroni-correct p-value < 0.0045, there was a statistically significant improvement in the neck by 55.9% (5.9 ± 0.6 vs 2.6 ± 0.4, p = 0.0001), shoulder & proximal arms by 66.0% (4.7 ± 1.1 vs 1.6 ± 0.5, p = 0.0034), and total dystonia by 48.6% (21.6 ± 3.7 vs 11.1 ± 2.1, p = 0.0001). There was a statistical significance using the uncorrected p-value < 0.05 in distal legs & feet by 47.2% (5.3 ± 1.9 vs 2.8 ± 1.1, p = 0.0310) and trunk dystonia by 89.8% (4.9 ± 1.1 vs 0.5 ± 0.5, p = 0.0069). As shown in Figure 3B, all 7 patients with trunk dystonia had improvement, 6 with >50% improvement and 1 with <50% improvement. Eleven out of 18 patients with neck dystonia and 10 out of 16 with dystonia in shoulder & proximal arms demonstrated >50% improvement post-DBS. In the following body regions, there were more patients who had no change or worse post-DBS sub-scores compared to the number of patients who demonstrated improvement: lower face (4 out of 7), jaw & tongue (7 out of 11), and larynx (4 out of 7).

## Discussion

This study evaluated response of dystonia by body distribution using both BFMRS-M and GDRS metrics after pallidal DBS. Neck dystonia showed significant improvement post-DBS using both BFMRS-M and GDRS, while shoulder & proximal arms also showed significant improvement when using GDRS. The overall findings in this study confirm a differential response to DBS by body distribution of dystonia. Incorporating this information into patient counseling and treatment decisions for DBS therapy may allow us to provide meaningful insight for our patients and help in addressing realistic expectations.

Notably, a large proportion of patients with speech & swallow dysfunction or laryngeal dystonia demonstrated a worsening score post-DBS. Although there was no statistically significant decrease in mean scores comparing baseline to post-DBS, this may be a clinically important consideration. The possible contributors to this finding include disease progression, surgical or stimulation-induced side effects, or limitations of video assessment.

We also demonstrate that, while BFMRS-M is currently used most often in existing literature in the field of dystonia, utilizing GDRS as an outcome measure may detect treatment response with higher quality and sensitivity. By applying GDRS, we show there is an improvement in proximal arm dystonia and possibly distal leg dystonia, but this is not sufficiently captured when applying BFMRS-M which scores the proximal and distal extremities together. This discrepancy is again apparent when comparing cranial dystonia. The BFMRS-M sub-score for mouth dystonia includes mouth, tongue, and jaw dystonia. On the other hand, GDRS scores lower face dystonia and jaw & tongue dystonia separately. There was no change in mean BFMRS-M for the mouth, while GDRS sub-scores for lower face improved (mean change –0.9) and GDRS sub-scores for jaw & tongue worsened (mean change +0.2). Our data highlights the discriminative limitations of BFMRS-M and that the GDRS was more sensitive in detecting significant outcomes in dystonia response.

Interestingly, 2 patients had worse total BFMRS-M scores compared to baseline, while all patients demonstrated improvement in total GDRS. This may be explained by the adjusted weighted calculations and incorporation of provoking factors in the BFMRS-M scale. When using BFMRS-M, Patient 2 had a worse post-DBS trunk sub-score compared to baseline (see Supplemental Table 1). The baseline BFMRS-M for this patient had a clinical severity of 3 (moderate bending causing gait impairment) and provoking factor of 2 (dystonia on many actions) yielding a sub-score of 6. The post-DBS BFMRS-M had a clinical severity of 2 (obvious bending but insufficient to impair gait) and provoking factor of 4 (dystonia present at rest) yielding a sub-score of 8. While the patient’s trunk sub-score worsened post-DBS due to a higher provoking factor, their dystonia improved such that it no longer caused clinical gait impairment. Given the limited ordinal scale (0–4) of BFMRS-M, clinically meaningful improvements following DBS were not adequately captured. Contrarily, this patient’s GDRS trunk sub-score improved from 8 at baseline to 3.5 post-DBS, which likely better reflects the treatment response (see Supplemental Table 2). When using GDRS, all patients with trunk dystonia showed an improvement post-DBS.

One proposed critique of GDRS is that physician experience may play a large role in attribution of points thus leading to high inter-rater variability. The BFMRS-M scoring sheet provides clear indications for each point scored on clinical severity as well as provoking factor while the GDRS scoring sheet instructs the physician to rate clinical severity from 0 to 10. However, in our study, the same five patients had sub-score discrepancies for both BFMRS-M and GDRS scores, requiring a third reviewer. Based on the limited data available in our study, GDRS rating did not have a higher inter-rater variability compared to BFMRS-M.

One of the major limitations of our analysis is our sample size. While a 20-patient cohort is larger than all other studies previously published on this topic, an even larger study size is necessary to improve power. A larger sample size in future work would allow for a more balanced representation of dystonia subtypes and stratified analysis by dystonia phenotype, age at onset, disease duration, or genetic status. Due to the retrospective nature of this study, genetic status was not known for all patients and active medications at the time of postoperative follow-up were not consistently available. Medication adjustments at various time points from DBS surgery and programming could impact exam scores. Another limitation is the variable postoperative follow-up interval, with a range from 6 to 30 months. This is particularly relevant given the timing of clinical response of dystonia to DBS which tends to be later than other indications for DBS and may also vary across body regions. Thus, a larger prospective study with standardized data collection of genetic status and medications and consistent postoperative follow-up interval may be helpful to validate these results.

Future considerations include obtaining patient-reported outcomes including quality-of-life data. Identifying any correlation between quality-of-life and BFMRS-M or GDRS may provide further insight into which motor scales have the most utility in research and clinical practice.

## Additional File

The additional file for this article can be found as follows:

10.5334/tohm.1175.s1 Supplementary File. Supplemental Tables 1 and 2.
