# Supplementary material for: Pallidal Deep Brain Stimulation in Dystonia: Investigating Differential Response by Dystonia Distribution
Source: Tremor Other Hyperkinet Mov (N Y). 2026 Jun 26;16:42. doi: 10.5334/tohm.1175 (PMC13308525; doi:10.5334/tohm.1175)
Supplement: Supplementary File. — Supplemental Tables 1 and 2. [file tohm-16-1-1175-s1.pdf]

	Eyes		Mouth		Speech & Swallow		Neck		Arms		Legs		Trunk		TOTAL	
	Pre	Post	Pre	Post	Pre	Post	Pre	Post	Pre	Post	Pre	Post	Pre	Post	Pre	Post
**1**					1.0	0.0	1.5	0.0	28.0	20.0	28.0	11.0	16.0	0.0	74.5	31.0
**2**			0.0	6.0	0.0	2.0	3.0	2.5	10.0	22.0	24.0	11.0	6.0	8.0	43.0	51.5
**3**	0.0	1.0			1.0	2.0	8.0	4.0	19.5	7.0	3.0	0.0	9.0	0.0	40.5	14.0
**4**									18.0	16.0	7.0	2.0	0.5	0.0	25.5	18.0
**5**			1.5	1.3	0.0	0.7	8.0	1.7	10.5	12.0	6.0	1.3			26.0	17.0
**6**	8.0	0.0	0.7	3.7			8.0	3.3	6.0	1.0					22.7	8.0
**7**					1.0	0.0	5.3	4.0	21.0	7.0					27.3	11.0
**8**							0.3	0.0	16.5	8.0					16.8	8.0
**9**			6.0	0.5	0.0	1.0	6.0	1.8	1.0	0.0					13.0	3.3
**10**	0.2	0.0	0.2	4.0			7.3	1.5	12.0	7.0					19.7	12.5
**11**			0.3	0.5	1.0	2.5	1.8	4.0	4.0	8.0	3.0	4.0			10.0	19.0
**12**			3.0	0.0	1.5	1.0	4.0	2.0	2.0	2.0					10.5	5.0
**13**	1.0	0.0	0.0	0.3	1.0	0.7	6.0	6.0	5.0	2.3			0.5	0.3	13.5	9.7
**14**	1.0	0.0	5.3	4.5	1.0	2.0	2.3	0.0	2.0	1.0					11.5	7.5
**15**			3.0	1.5			6.0	4.0							9.0	5.5
**16**							6.0	4.0	4.0	0.0	0.5	0.0			10.5	4.0
**17**			2.3	0.2	2.7	3.3	6.0	5.3					0.7	0.0	11.7	8.8
**18**	0.0	0.3					8.0	0.0	0.5	0.0					8.5	0.3
**19**							0.0	0.3					9.0	0.0	9.0	0.3
**20**					0.0	1.0	6.0	3.5	0.5	0.0					6.5	4.5
**Mean**	**1.7**	**0.2**	**2.0**	**2.0**	**0.8**	**1.3**	**4.9**	**2.5**	**9.4**	**6.7**	**10.2**	**4.2**	**6.0**	**1.2**	**20.5**	**11.9**

Supplemental Table 1: Patient-level BFMRS-M scores at baseline (pre) and post-DBS (post). Scores are left blank for patients with baseline sub-score 0 and post-DBS sub-score 0 in a particular body region, as these patients were excluded from the data analysis of that body region.

	Eyes		Lower Face		Jaw & Tongue		Larynx		Neck		Shoulder & Proximal Arms		Distal Arms & Hands		Pelvis & Proximal Legs		Distal Legs & Feet		Trunk		TOTAL	
	**Pre**	**Post**	**Pre**	**Post**	**Pre**	**Post**	**Pre**	**Post**	**Pre**	**Post**	**Pre**	**Post**	**Pre**	**Post**	**Pre**	**Post**	**Pre**	**Post**	**Pre**	**Post**	**Pre**	**Post**
**1**							1.0	0.0	2.0	0.0	17.5	5.5	15.5	12.0	17.0	9.0	15.5	9.0	8.5	0.0	77.0	35.5
**2**					0.0	5.0			4.5	4.5	9.0	5.0	0.5	12.0	20.0	0.0	12.5	7.0	8.0	3.5	54.5	37.0
**3**	0.0	1.0					0.0	2.0	9.0	3.0	4.5	4.5	13.5	3.5			3.0	0.0	5.5	0.0	35.5	14.0
**4**											7.5	0.5	7.5	10.0	4.5	0.0	6.5	3.0	3.5	0.0	29.5	13.5
**5**			1.5	2.0	0.0	2.0			9.0	2.7	1.0	1.0	5.5	6.3	2.5	0.0	2.0	1.0			21.5	15.0
**6**	9.0	0.0	0.0	1.3	0.0	3.3			9.0	2.7	3.3	0.0									21.3	7.3
**7**							1.5	0.0	7.0	5.0	2.0	0.0	7.5	6.5							18.0	11.5
**8**											10.0	0.0	8.0	4.0							18.0	4.0
**9**			5.5	1.0	5.5	1.0			5.5	1.5			1.0	0.0							17.5	3.5
**10**	0.3	0.0			0.3	4.0			7.3	2.5	7.3	4.5	2.0	3.0							17.3	14.0
**11**					2.5	2.5			2.0	2.0	4.5	1.0	2.0	5.0	3.5	3.5	2.0	2.0			16.5	16.0
**12**			3.0	0.0	2.5	0.0	2.5	1.0	5.0	2.0	2.5	0.0	0.5	2.5							16.0	5.5
**13**	2.0	0.0	0.0	0.7	0.0	0.7	0.0	1.0	7.5	5.3	2.0	2.0	3.0	0.3			0.0	0.3	0.5	0.3	15.0	10.7
**14**	1.5	0.0	4.0	5.5	4.5	4.5			2.5	0.0			2.0	1.0							14.5	11.0
**15**			3.5	1.0	2.0	0.0			8.0	2.5	0.0	1.0									13.5	4.5
**16**									7.0	3.5	2.0	0.0	2.5	0.0			0.5	0.0			12.0	3.5
**17**					3.3	0.3	0.0	3.7	6.3	5.3									1.7	0.0	11.3	9.3
**18**	0.0	0.5							9.0	0.0	2.0	0.0									11.0	0.5
**19**									0.0	0.5									6.5	0.0	6.5	0.5
**20**							0.0	1.0	6.0	4.5	0.5	0.0									6.5	5.5
**Mean**	**2.1**	**0.3**	**2.5**	**1.6**	**1.9**	**2.1**	**0.7**	**1.2**	**5.9**	**2.6**	**4.7**	**1.6**	**5.1**	**4.7**	**9.5**	**2.5**	**5.3**	**2.8**	**4.9**	**0.5**	**21.6**	**11.1**

Supplemental Table 2: Patient-level GDRS scores at baseline (pre) and post-DBS (post). Scores are left blank for patients with baseline sub-score 0 and post-DBS sub-score 0 in a particular body region, as these patients were excluded from the data analysis of that body region.
